# Antiproliferative Effects of Fluoxetine on Colon Cancer Cells and in a Colonic Carcinogen Mouse Model

**DOI:** 10.1371/journal.pone.0050043

**Published:** 2012-11-27

**Authors:** Vinicius Kannen, Henning Hintzsche, Dalila L. Zanette, Wilson A. Silva, Sérgio B. Garcia, Ana Maria Waaga-Gasser, Helga Stopper

**Affiliations:** 1 Department of Pathology, Medical School of Ribeirão Preto, University of São Paulo, Ribeirão Preto, Brazil; 2 Department of Toxicology, University of Wuerzburg, Wuerzburg, Germany; 3 National Institute of Science and Technology in Stem Cell and Cell Therapy, CNPq/FAPESP, Department of Genetics, Medical School of Ribeirão Preto, University of São Paulo, Ribeirão Preto, Brazil; 4 Department of Surgery I, Molecular Oncology and Immunology, University of Wuerzburg, Wuerzburg, Germany; University of Patras, Greece

## Abstract

The antidepressant fluoxetine has been under discussion because of its potential influence on cancer risk. It was found to inhibit the development of carcinogen-induced preneoplastic lesions in colon tissue, but the mechanisms of action are not well understood. Therefore, we investigated anti-proliferative effects, and used HT29 colon tumor cells *in vitro*, as well as C57BL/6 mice exposed to intra-rectal treatment with the carcinogen N-methyl-N’-nitro-N-nitrosoguanidine (MNNG) as models. Fluoxetine increased the percentage of HT29 cells in the G_0_/G_1_ phase of cell-cycle, and the expression of p27 protein. This was not related to an induction of apoptosis, reactive oxygen species or DNA damage. *In vivo*, fluoxetine reduced the development of MNNG-induced dysplasia and vascularization-related dysplasia in colon tissue, which was analyzed by histopathological techniques. An anti-proliferative potential of fluoxetine was observed in epithelial and stromal areas. It was accompanied by a reduction of VEGF expression and of the number of cells with angiogenic potential, such as CD133, CD34, and CD31-positive cell clusters. Taken together, our findings suggest that fluoxetine treatment targets steps of early colon carcinogenesis. This confirms its protective potential, explaining at least partially the lower colon cancer risk under antidepressant therapy.

## Introduction

Colon cancer is one of the major human malignancies worldwide, and much effort has been applied to understand the process of colon carcinogenesis, as well as the role of potential treatments and co-therapeutical agents against it [1]–[3]. A growing body of evidence suggests that the use of fluoxetine (FLX), an antidepressant belonging to the selective serotonin reuptake inhibitors (SSRIs), may be associated with a reduced colon cancer risk [4]–[6]. However, controversial opinions have been published [7]–[13] and an identification of the mechanisms of the activity of FLX on colon cells would help in the clarification of this controversy.

We recently found that FLX reduced the number of 1,2 dimethylhydrazine (DMH)-induced preneoplastic colonic lesions, termed aberrant crypt foci (ACF) and exerted an early anti-VEGF activity on stromal cells, decreasing microvessel numbers within pericryptal colonic stroma (PCCS) [5]. Tumor stroma constitutes 60–90% of the colon tumor mass [14], and PCCS surrounding the cryptal bottom has been reported to initiate certain steps in colon tumor development, such as increased proliferation, microvessel formation, VEGF-synthesis, regulation of self-renewal and differentiation of intestinal cells [15]–[18]. ACFs are considered a suitable experimental model for studying early stages of colon cancer formation, mainly due to their close resemblance with the development of cancer in rodents and humans [17], [19].

**Figure 1:**
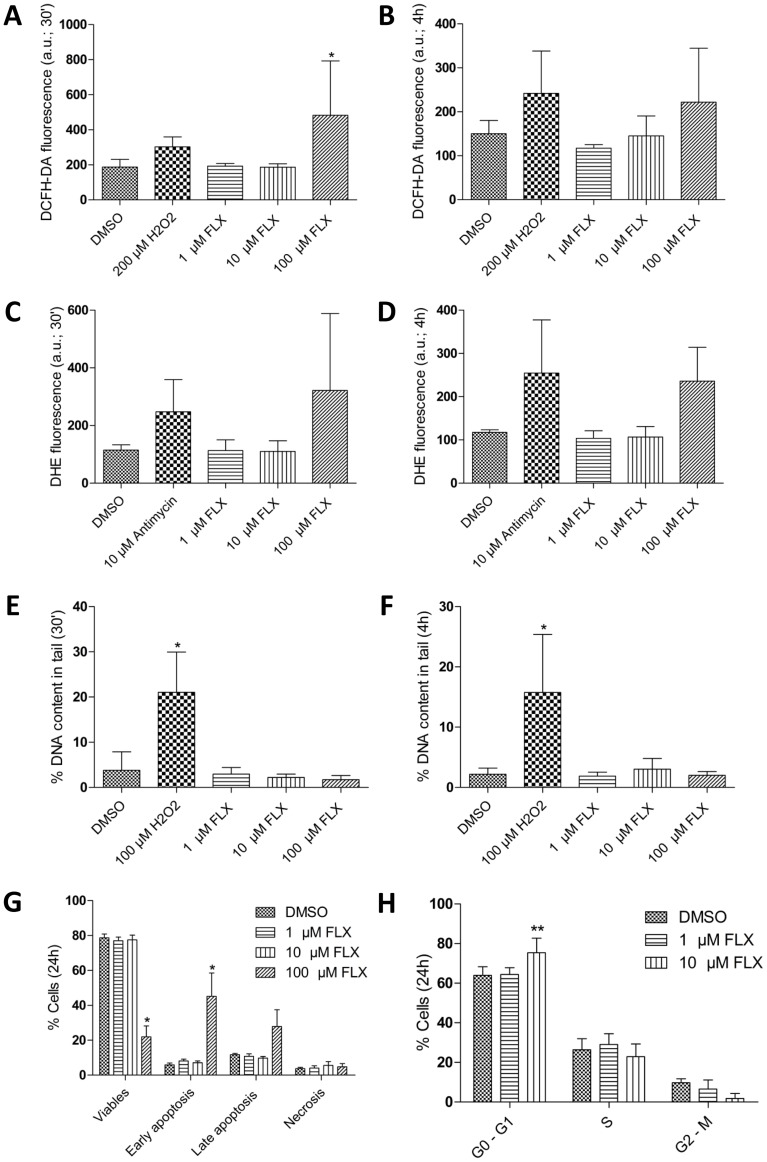
Influence of Fluoxetine (FLX) on reactive oxygen species (ROS) production, DNA-damage, cell vitality/apoptosis and cell-cycle progression in HT29 cells. (A and B) ROS production analyzed by flow cytometry, after exposure to different FLX concentrations for 30 min (A), and 4 h (B; *P<0.05 *vs* DMSO). Hydrogen peroxide (H_2_O_2_) was used as positive control and in both cases applied for 30 min; arbitrary units (a.u.). (C and D) O_2_^−^ production detected by DHE staining for 30 min (C; P>0.05 *vs* DMSO), and 4 h (D; P>0.05). (E and F) DNA-damage analyzed by comet assay, after exposure to different FLX concentrations for 30 minutes (E), and 4 h, with % DNA content in tail representing DNA-damage (F; *P<0.05 *vs* DMSO). Hydrogen peroxide (H_2_O_2_) was used as positive control and in both cases applied for 30 min. (G) Viability and apoptosis assay (Annexin V/PI) performed by flow cytometry, after exposure to different FLX concentrations for 24 hours (^*^P<0.05 *vs* DMSO). (H) Distribution of cells in cell cycle phases analyzed by flow cytometry. Given is the percentage of HT29 cells in G_0_/G_1_, S, and G2/M phases with and without FLX treatment for 24 h (**P<0.01 vs DMSO). All figures show results from at least 4 independent experiments.

Although FLX is known to decrease colon cell proliferation *in vitro* [20], and *in vivo* models [5], [21], the mechanism of its antiproliferative activity is not well understood. Here we investigated whether antiproliferative effects of FLX treatment play a role in the chemoprevention of dysplasia in colon tissue and what the involved mechanisms are. For this purpose, we used a human colon cancer cell line (HT29) for *in vitro* experiments and an *in vivo* model for studying carcinogen-induced preneoplastic lesions. The *in vivo* model consisted of C57BL/6 mice exposed to intra-rectal treatment with the alkylating mutagen and carcinogen N-methyl-N’-nitro-N-nitrosoguanidine (MNNG). MNNG has been reported to induce ACFs [22] to higher degree than 1,2 dimethylhydrazine (DMH), the carcinogen previously used in colon cancer models by our group [5], [17], [23].

Our results show that the antiproliferative effects of FLX-treatment were not induced by increased apoptosis or production of reactive oxygen species (ROS) or DNA damage. Instead FLX reduced ACF numbers, most likely by controlling the activity of stromal cells related to microvessel development.

**Figure 2:**
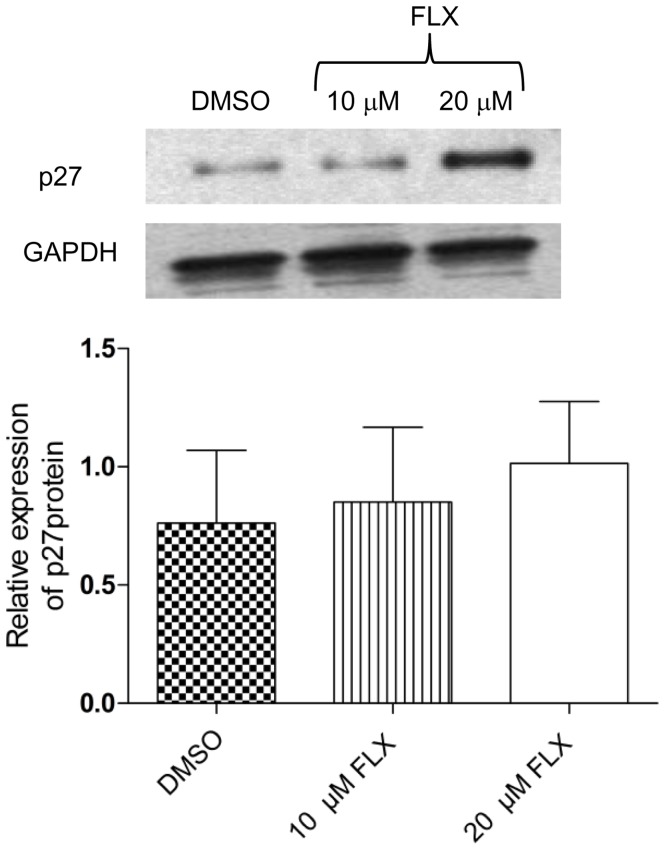
Effect of Fluoxetine (FLX) on p27 protein levels. Relative expression of p27 protein in HT29 cells as determined by Western blot analysis (P>0.05 *vs* DMSO). A sample blot and the average result of 4 independent experiments are shown.

## Materials and Methods

### Reagents

Fluoxetine, tempol, hydrogen peroxide (H_2_O_2_), dimethyl sulfoxide (DMSO), bisbenzimide, DAPI (4′,6-diamidino-2-phenylindole), and MNNG were purchased from Sigma-Aldrich (Louis, MO, USA). DMEM (4.5 g/L glucose) medium were purchased from PAA Laboratories GmbH (Austria). Dihydroethidium (DHE) was acquired from Merck Millipore (Germany). Dichlorofluorescein (DCFH-DA) and Annexin V apoptosis detection kit (FITC), and Cytofix/Cytoperm kit were acquired from Becton Dickinson (Germany).

### Cell Culture

The HT29 human colon cancer cell line was obtained from the American Type Culture Collection (Manassas, VA, USA) and cultured under standard conditions and grown in DMEM (4.5 g/L glucose) medium. It was supplemented with 10% FBS, 1% L-glutamine, penicillin (100 units/mL) and 0.1 mg/ml streptomycin. Cells were seeded into 6-well plates (Sarstedt Inc., USA), at an initial concentration of 5×10^5^ cells/well, and left untreated for 20–24 hours. Then cells were exposed to 1, 10 or 100 µM FLX for 30 minutes, 4 or 24 hours. H_2_O_2_ (100 or 200 µM) served as control for oxidative effects and DNA damage. Cells were exposed to H_2_O_2_ for 30 minutes.

### Flow Cytometry for Oxidative Stress, Viability and Apoptosis, and Cell-cycle Analysis

Analysis for reactive oxygen species (ROS) production was carried out in accordance with our standard method by flow cytometry and argon laser excitation [24]. Briefly, cells were labeled for 10 minutes with DCFH-DA and, then analyzed with a FL1 band pass filter (Becton Dickinson [BD] LSR I™, Germany). Superoxide was detected after staining cells for 30 minutes with DHE (10 µM) in medium without serum. After harvesting, cells were analyzed with a FL2 band pass filter [25]. A viability and apoptosis assay was performed by an Annexin V/Propidium iodide (PI) kit and analyzed with FL1 and FL2 band pass filters, according to the manufacturer’s instructions. For cell cycle analysis, HT29 cells were incubated with FLX (1 µM and 10 µM) or the solvent DMSO (1%). Subsequently, cells were permeabilized (Cytofix/Cytoperm kit) and stained with bisbenzimide for 30 min. Experiments were analyzed with ultraviolet (UV) laser excitation and a FL5 band pass filter. 20,000 cells were analyzed per sample and evaluated with BD CellQuest Pro™ software. Cell cycle phases were analyzed by ModFit LT ™ software (Verity Software House, USA). At least four independent experiments were carried out.

**Figure 3:**
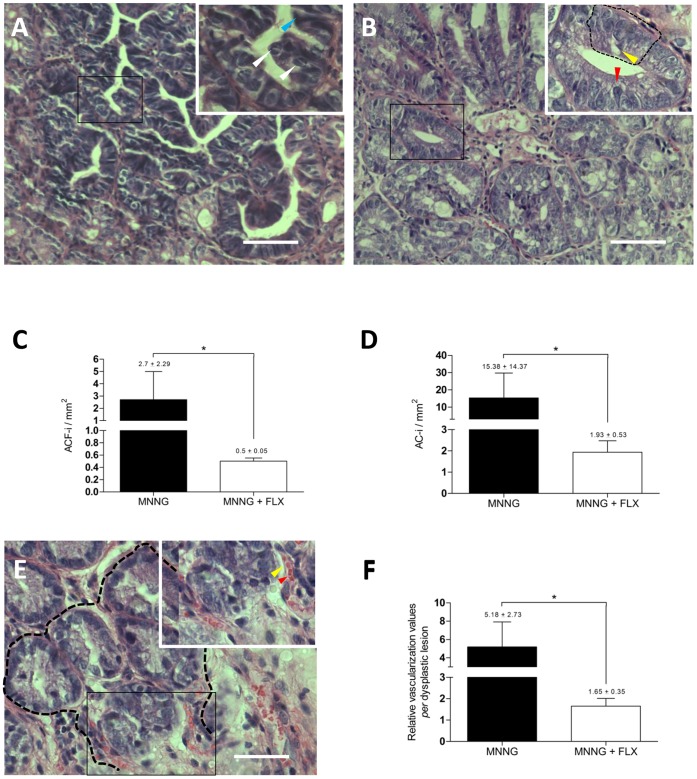
Chemopreventive activity of fluoxetine in colon tissue. (**A**) Representative histological image of a severely dysplastic area (MNNG-exposed mouse). The picture inset (enlarged from the boxed region) shows the characteristic severe dysplastic features; e.g., partial loss of cell polarity, none goblet cells, presence of Paneth cells (blue arrow), and mitosis (white arrows). Pictures were taken at 200x magnification, scale bars represent 20 µm. High-magnification images were taken at 1000x magnification. (**B**) Representative image of a moderate dysplasia (MNNG+FLX treated mouse). The inset shows a compressed cryptal luminal opening, elongated nuclei (red arrow), a crowded and pseudostrafied area (sectioned black line), but with a generally still preserved cell polarity, and a lower number of globet cells (yellow arrow). Magnifications are described above. (**C**) Quantification of dysplastic lesions. Aberrant crypt foci index (ACF-i) shown as number of dysplastic lesions *per* µm^2^ (*P<0.05; MNNG without FLX, *n* = 5; FLX+MNNG, *n = *4). (**D**) Aberrant crypt index (AC-i) shown as number of dysplastic single crypts *per* µm^2^ (*P<0.05; MNNG without FLX, *n* = 5; FLX+MNNG, *n = *4). (**E**) Representative image of a dysplastic area (sectioned black line), and its relative vascularization spreading inside. The inset (enlarged from the boxed region) shows two microvessels towards the inner region of the dysplastic area. The yellow arrow points to a microvessel wall, and the red arrow to erythrocytes inside the microvessel walls. Pictures were taken at 400x magnification, and further details are described above. (**F**) Relative dysplastic vascularization, shown as the number of microvessels *per* lesion (*P<0.05; MNNG without FLX, *n* = 5; FLX+MNNG, *n = *4).

**Figure 4:**
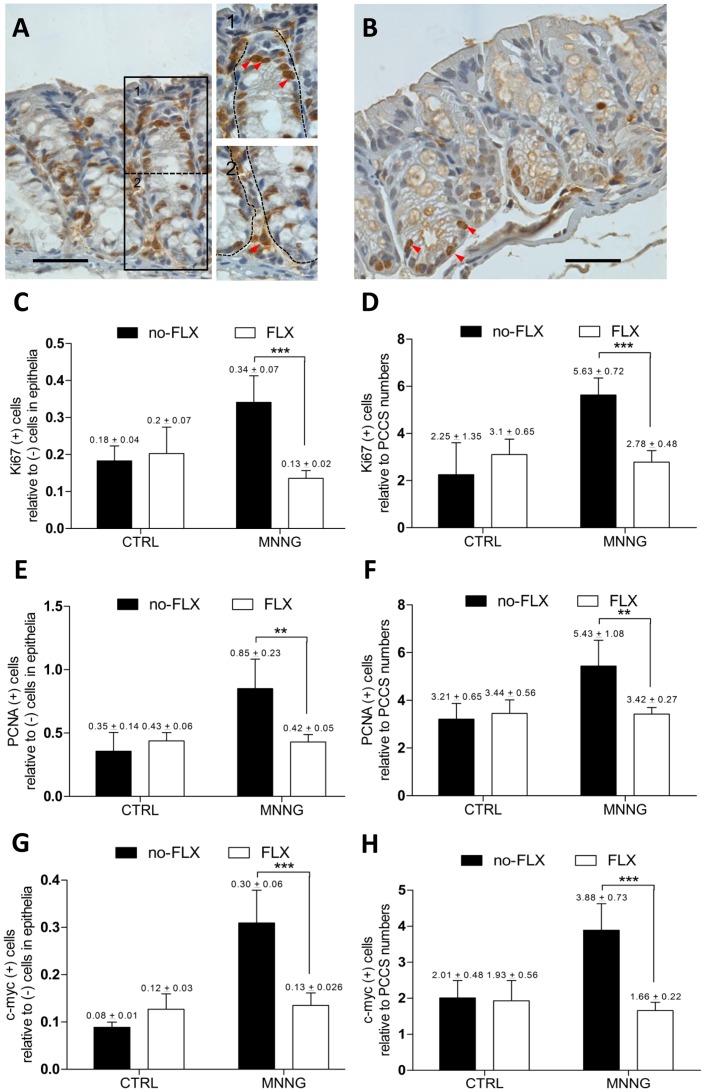
Antiproliferative activity of fluoxetine in colon tissue. (**A**) Proliferating cells detected by staining with anti-Ki67 antibody (MNNG-treated mouse). (1) Red arrows show dark-brown positive cells migrating upward in the epithelial proliferative zone. (2) Stromal positive cells are shown by a red arrow near to the cryptal-bottom. Pictures were taken as described above. (**B**) Proliferating cells (anti-Ki67 antibody; red arrows) in a MNNG+FLX-treated mouse are shown at the cryptal bottom. Pictures were taken as described above. (**C**) Proliferation in epithelial areas shown by labeling with anti-Ki67 antibody (***P<0.001; MNNG without FLX, *n* = 5; FLX+MNNG, *n = *4). (**D**) Proliferation in PCCS areas by labeling with anti-Ki67 antibody (***P<0.001; MNNG without FLX, *n* = 5; FLX+MNNG, *n = *4). (**E**) Proliferation in epithelial areas shown by labeling with anti-PCNA antibody (**P<0.01; MNNG without FLX, *n* = 4; FLX+MNNG, *n = *4). (**F**) Proliferation in PCCS areas shown by labeling with anti-PCNA antibody (**P<0.01; MNNG without FLX, *n* = 4; FLX+MNNG, *n = *4). (**G**) Expression of c-Myc in epithelia of colon tissue (***P<0.001; MNNG without FLX, *n* = 3; FLX+MNNG, *n = *4). (**H**) Expression of c-Myc in PCCS areas of colon tissue (***P<0.001; MNNG without FLX, *n* = 3; FLX+MNNG, *n = *4).

### Comet Assay

According to our previous description [24], an alkaline version of the comet assay was performed. The analysis encompassed 100 randomly selected cells (50 per replicate slide) for each sample, which were analyzed with a fluorescence microscope (Labophot 2, Nikon, Germany) at 200-fold magnification using Komet 5 image-analysis software (BFI Optilas, Germany). The percentage of DNA in the tail (% Tail DNA) was used to quantify DNA migration. At least four independent experiments were carried out.

### Western Blot Analysis

It was performed as described in NuPAGE Technical Guide (Invitrogen, USA). Briefly, protein extracts were run on NuPAGE 4–12% Bis-Tris Mini Gels (Invitrogen), and transferred to membranes with iBlot Dry Blotting System (Invitrogen).The membranes were incubated 4°C overnight with anti-p27 (D69C12, Cell Signaling, USA), and anti-GAPDH (9484, Abcam, UK) antibodies. Secondary antibodies (goat anti-rabbit and goat anti-mouse IgG HRP antibodies) were incubated 1 h at room temperature. Bands of labeled-antibodies were detected by using SuperSignal West Pico Chemiluminescent Substrate (Thermo Scientific, USA). Films were scanned and intensity of bands quantified with ImageJ software. Data from 4 independent experiments are shown as ratios between values from targeted and endogenous control proteins.

**Figure 5:**
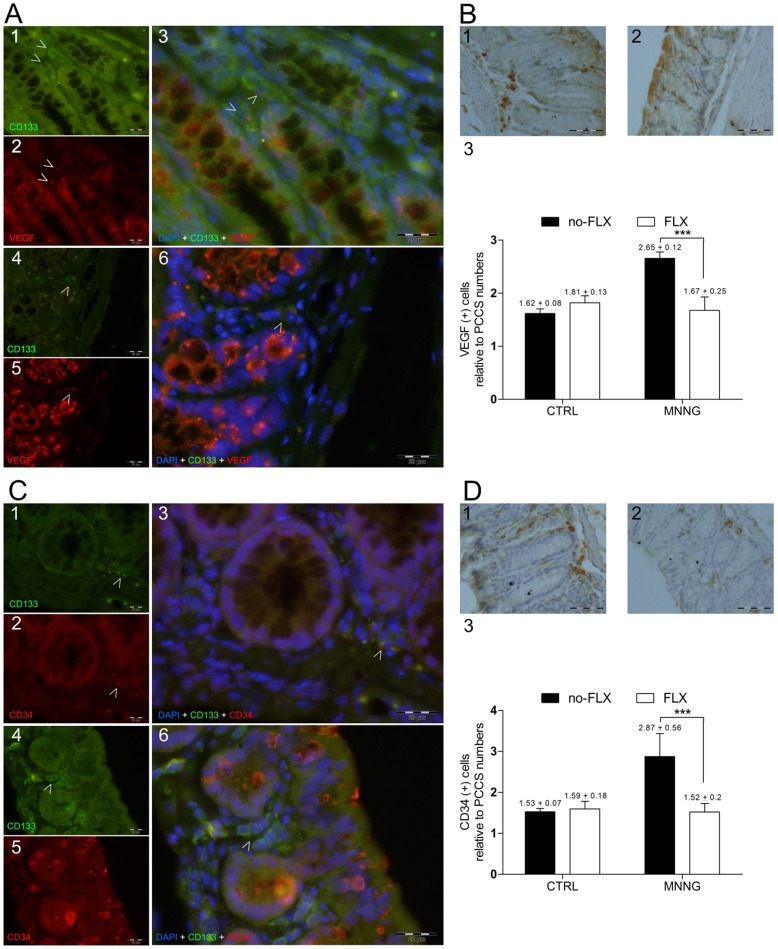
Fluoxetine activity and angiogenic-related markers. (**A**) Representative images of colon sections labeled with anti-CD133 and anti-VEGF antibodies, and nuclei stained with DAPI. White arrows indicate single-stained and double-stained positive cells into PCCS areas in colon sections from (**A1** to **A3**) MNNG without FLX treatment, and (**A4** to **A6**) MNNG+FLX treatment groups. Pictures were taken with FITC (495–521 nm), ultraviolet (358–461 nm), and Texas Red (595–605 nm) filters. All pictures were taken at 600x magnification, scale bars represent 20 µm. (**B**) Representative images of single colon-stained sections with anti-VEGF antibody (positive cells are seen with dark-brown cytoplasm) from (**B1**) MNNG without FLX treatment, and (**B2**) MNNG+FLX treatment groups. (**B3**) Relative number of cells expressing VEGF within PCCS areas (***p<0.001; MNNG without FLX, *n* = 3; FLX+MNNG, *n = *4). All pictures were taken at 400x magnification, scale bars represent 50 µm. (**C**) Representative images of colon sections labeled with anti-CD133 and anti-CD34 antibodies, and nuclei stained with DAPI. White arrows indicate single-stained and double-stained positive cells into PCCS areas in colon sections from (**C1** to **C3**) MNNG without FLX treatment, and (**C4** to **C6**) MNNG+FLX treatment groups. Pictures were taken as described above. (**D**) Representative images of single colon-stained sections with anti-CD34 antibody (positive cells are seen with dark-brown cytoplasm) from (**D1**) MNNG without FLX treatment, and (**D2**) MNNG+FLX treatment groups. (**D3**) Relative number of CD34 positive cells in PCCS areas (***p<0.001; MNNG without FLX, *n* = 4; FLX+MNNG, *n = *4). All pictures were taken as described above.

### Mice and Treatment Protocol

Female C57BL/6 mice (5 weeks) were supplied by the Medical School of Ribeirao Preto, University of Sao Paulo, Brazil. All *in vivo* treatments were in agreement with the protocol approved by the Animal Care and Use Committee (n° 068/2012) from the Medical School, University of São Paulo, and the guidance for animal managing at a minimum acceptable number. Mice were acclimated for 1 week before starting the experiment. C57BL/6 mice were exposed or not to the carcinogen MNNG and randomly assigned to one of four groups, as control (CTRL) or MNNG-treatment (four successive doses of MNNG [5 mg/ml; intrarectal deposits of 100 µl; Sigma-Aldrich, Louis, MO, USA] twice a week for 2 weeks), and FLX-treatment (30 mg/kg/day; intraperitoneal, i.p.; Sigma-Aldrich, Louis, MO, USA) or MNNG+FLX-treatment. Each group had five mice, and they were housed *per* plastic cage at 22±2°C with 55% humidity and 12 h light/dark cycle. FLX-application was started after 2 weeks from the end of MNNG-treatment, and continued for the next 4 weeks. All animals had free access to chow and water during the experiment. All mice were euthanized by CO_2_ exposure at week 8 of the experiment. Individual autopsies were performed, and colon tissue samples were longitudinally opened and fixed flat in 4% neutral paraformaldehyde-buffer (24 h). Mice with fragmented tissue sections were discarded from the analysis.

**Figure 6:**
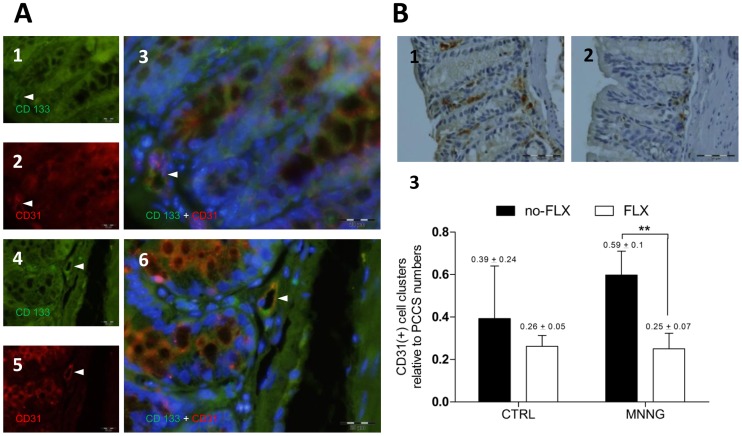
Fluoxetine activity and an angiogenic-related marker. (**A**) Representative images of colon sections labeled with anti-CD133 and anti-CD31 antibodies, and nuclei stained with DAPI. White arrows indicate single-stained and double-stained positive cells into PCCS areas in colon sections from (**A1** to **A3**) MNNG without FLX treatment, and (**A4** to **A6**) MNNG+FLX treatment groups. (A3 and A6) Double-stained positive cells are shown by white arrows in microvessel-like structures nearby cryptal bottoms. Pictures were taken with FITC (495–521 nm), ultraviolet (358–461 nm), and Texas Red (595–605 nm) filters. All pictures were taken at 600x magnification, scale bars represent 20 µm. (**B**) Representative images of single colon-stained sections with anti-CD31 antibody (positive cells are seen with dark-brown cytoplasm) from (**B1**) MNNG without FLX treatment, and (**B2**) MNNG+FLX treatment groups. Pictures were taken at 400x magnification, scale bars represent 50 µm. (**B3**) Relative number of CD31 positive cell clusters detected *per* PCCS area (**p<0.01; MNNG without FLX, *n* = 4; FLX+MNNG, *n = *4).

### Histopathological Analysis

Colon tissue samples were sectioned and stained with H&E and analyzed using light microscopy. A tissue overview was carried out at 200x magnification in colonic samples, where dysplastic aberrant crypt foci (ACF) with pathological features ranging from mild to severe dysplasia were detected and counted [26]. Afterwards, a second analysis was carried out at 400x magnification on each detected lesion for confirmation of dysplastic features and counting the number of aberrant crypts (AC) and microvessels (MV). The whole area of each analyzed section was determined with a graduated lens (100x*;* Nikon, Japan), and its area (mm^2^) was calculated as *values (V) × 0.9801/121*. Relative values for ACF-i (index) and AC-i were calculated as their total number *per* mm^2^ [5], [17]. The vascularization-related dysplasia was determined to be *ACF × MV/AC*.

### Immunohistochemistry (IHC) and Immunofluorescence (IMF)

According to previous description [5], [27], IHC and IMF staining were performed on paraffin colonic sections (4 µm). Primary antibodies were purchased from Novocastra (USA), Santa Cruz Biotechnology (Germany), and Biocare Medical (USA). Sections were incubated with anti-Ki67 (clone MMA at 1∶100), anti-PCNA (clone PC 10 at 1∶100), anti-c-*Myc* (clone 9E11 at 1∶100), anti-VEGF (clone A-20 at 1∶100), anti-CD34 (clone QBEnd/10 at 1∶100), anti-CD31 (clone 1A10 at 1∶100), and anti-CD133 (clone N/A at 1∶100) primary antibodies overnight. The brown color was displayed by incubating sections with Picture-MAX Polymer Kit (Invitrogen, USA). It showed in positive reactions a brown precipitate at the nucleus for Ki67, and PCNA, and in the cytoplasm and/or perinuclei area for *c-Myc*, VEGF, CD133, CD34, and CD31.

Proliferation in colonic sections was analyzed with anti-Ki-67 and anti-PCNA antibodies in epithelial and PCCS areas. Markers associated with vascularization (VEGF, CD133, CD34, and CD31) were counted in PCCS areas. However, CD31 (or PECAM-1; platelet endothelial adhesion molecule-1) was counted as positive cell clusters (more than 3 positive cells), since CD31 is mainly expressed on the surface of endothelial cells [28]. Ratios from counting were determined between positively stained nuclei and total unstained nuclei in epithelia, whereas ratios in PCCS areas were calculated between positive cells (or CD31 clusters) and the total number of counted areas. For positive cells *per* cluster of CD31, the ratio was calculated between the number of labeled cells and the total number of clusters.

Double-labeling was performed in fixed colon samples labeled with mouse anti-human CD133 (1∶100; Miltenyl Biotec, 715-090-422; secondary anti-mouse FITC conjugated antibody, 1∶400, Dianova, 715-095-150), rabbit anti-mouse VEGF (1∶100, Santa Cruz, sc-152; secondary anti-rabitt Cy3 conjugated antibody, 1∶400, Dianova, 111-165-144), rat anti-mouse CD34 (1∶100, Applied Biosystems, ab 8158; secondary anti-rat Texas Red conjugated antibody, 1∶400, GeneTex, GTX 26732), and rabbit anti-mouse CD31 (1∶100, Applied Biosystems, ab 28365; secondary anti-rabitt Cy3 conjugated antibody, 1∶400, Dianova, 111-165-144) antibodies. Nuclei were stained with DAPI. Images were acquired with an Olympus BX51 microscopy equipped with a Olympus DP71 camera and a CellSens Dimension software (Olympus, Germany).

### Statistical Analysis

Data were analyzed using the statistical program GraphPad Prism 5.0 (Graph Pad Software Inc., San Diego, California, USA). Two-way ANOVA (Bonferroni post hoc test) test was applied to analyze data from annexin V/PI and cell-cycle assays, and *in vivo* experiments, since it allows different endpoints to be analyzed separately. Oxidative stress and DNA damage were analyzed by One-way ANOVA (Bonferroni post hoc test) test. Preneoplastic lesions (ACF-i, AC-i, and vascularization-related dysplasia) were analyzed by Mann Whitney test. A probability of P<0.05 was considered to be statistically significant. All values represent means±standard deviations.

## Results

### FLX Effects on ROS Production, DNA Damage, Cell Viability, Apoptosis, and Cell-cycle

To understand whether and how therapeutic and over-therapeutic concentrations of FLX could play a role against colon cancer cell proliferation, human colon cancer HT29 cells were analyzed for ROS production, DNA damage, viability, apoptosis, and the distribution of cell-cycle phases in the culture. We found that ROS production was increased 2.5-fold in HT29 cells by 100 µM FLX after 30 min treatment, but no significant increase was observed with 1 and 10 µM FLX (Figure 1A). Further experiments showed that ROS were about 2-fold less in HT29 cells exposed to 100 µM FLX after 4 h than after the 30 min treatment (Figure 1B). With the more superoxide specific dye DHE a similar pattern was observed (Figure 1C and 1D). A 2.8-fold increase with 100 µM FLX (Figure 1C), but no increase with 1 µM and 10 µM FLX was found after 30 min and a 2-fold enhancement was observed after 4 h (Figure 1D), again only with 100 µM FLX. Thus, the increase with 100 µM FLX was 1.4-fold less after 4 h treatment than after 30 min. None of the FLX concentrations induced significant DNA damage in HT29 cells after 30 min or 4 h experiments (Figure 1E and F).

After 24 h, 100 µM FLX decreased cell viability significantly and induced apoptosis, although 1 and 10 µM were unable to elicit similar effects (Figure 1G). On the other hand, 10 µM FLX caused a significant delay of cells in the G_0_/G_1_ cell-cycle phase during a 24 h treatment (Figure 1G). When the cell cycle progression related protein p27 was investigated, 10 µM FLX caused a slight upregulation and a 1.3-fold increase was found after 24 h treatment with 20 µM FLX (Fig. 2). Taken together, a G_0_/G_1_ cell-cycle delay occurred at a therapeutical FLX concentration, which was not caused by ROS production or induction of DNA damage.

### Potential of FLX Against the Formation of Preneoplastic Lesions

Based on the findings shown above, a chemopreventive activity against preneoplastic lesions in colon tissue was verified for FLX treatment in carcinogen-exposed mice. Mice were first exposed to MNNG, treated with FLX for 28 days, and preneoplastic lesions were enumerated by histopathological analysis. We observed severe MNNG-induced dysplasia (Figure 3A) in colonic sections, which was not observed to such a grade in samples from FLX-treated animals (Figure 3B). Quantification of dysplastic lesions (ACF) in MNNG and MNNG+FLX-treated mice showed that FLX reduced dysplasia 5.39-fold (Figure 3C). FLX treatment significantly reduced the total values of AC *per* mm^2^ 7.94-fold (Figure 3D). Microvessels were spread throughout PCCS towards dysplastic areas (Figure 3E), and we found that FLX treatment decreased the vascularization-related dysplasia 3.13-fold (Figure 3F). Our data suggest that FLX acts in different colonic areas, namely epithelia and stroma.

### Activity of FLX on Proliferation in Epithelia and PCCS Areas

Since we indentified chemopreventive effects under FLX-treatment in colon tissue, the question was raised whether these findings were related to antiproliferative activities in two different colonic areas, namely epithelia and PCCS areas (Figure 4A.1 and 2). Labeled sections with anti-Ki67 antibody revealed that FLX attenuated (Figure 4B) the MNNG-induced increase in proliferation, at epithelial and PCCS areas (Figure 4C and D). PCNA staining also showed that FLX attenuated the MNNG-induced proliferative activity at both colonic areas (Figure 4E and F). Furthermore, a high *c-Myc* expression was found at both colonic areas in MNNG-treated animals, in which FLX-treatment prevented the increase of expression (Figure 4G and H). Thus, our findings support the hypothesis that inhibition of proliferation plays an important role in the chemopreventive effects of FLX treatment.

### Activity of FLX on Angiogenesis

Considering that angiogenesis takes place within PCCS, and proliferation as well as stem cell markers might be related to that [15]–[16], [29]–[30], we investigated the potential connection between these events under FLX treatment. CD133-positive cells expressing VEGF were found within PCCS in mice subjected either to MNNG or MNNG+FLX treatments (Figure 4A). FLX treatment decreased their relative number in the carcinogen-exposed group compared to the MNNG group without FLX (MNNG, 3.73±0.44 [*n* = 4] *vs* MNNG+FLX, 1.97±0.14 [*n* = 4]; P<0.001). Stromal cells expressing VEGF were also decreased in MNNG-exposed animals treated with FLX expression (Figure 5B). Intriguingly, CD133 positive cells expressing CD34 glycoprotein were only found among MNNG treated mice (Figure 5C) while FLX treatment significantly decreased the number of stromal CD34-positive cells (Figure 5D). Further, the relative total number of CD31-positive cells was enumerated within PCCS areas, and a significant decrease was found in the MNNG-exposed group under FLX treatment (MNNG, 3.5±0.87 [*n* = 4] *vs* MNNG+FLX, 1.8±0.2 [*n* = 4]; P<0.01). A comparison between CD34 and CD31-positive cells showed a 1.2-fold increase in CD31-positive cell values among MNNG-exposed mice.

Since CD31 is an angiogenesis-related marker [31] and was enhanced under MNNG-treatment, double-staining was performed to understand whether CD133 positive cells were expressing CD31 glycoprotein. In the MNNG and MNNG+FLX groups, CD133 positive cells expressed CD31 in microvessel-like structures within PCCS areas (Figure 6B). Potential sites of developing microvessels were detected enumerating CD31-positive cell clusters within PCCS (Figure 6B.1 and B.2). CD31-positive cell clusters were decreased significantly under FLX-treatment in MNNG-exposed mice (Figure 6B.3). Therefore, these data lead to the hypothesis that a reduction of microvessel formation might be occurring due to the control of FLX upon the stromal cell differentiation process.

## Discussion

Therapeutical concentrations of FLX range from 10 to 30 µM in the human brain [32]. This concentration (10 µM) delayed cell-cycle progression independently of ROS production and DNA-damage in human colon cancer cells *in vitro*. Similar findings of FLX blocked cell cycle progression arresting breast tumors cells at G_o_/G_1_ phase have been reported previously [33]. These authors found among other evidence an accumulation of p27 and developed a modeling-based hypothesis that FLX can disrupt the assembly of cyclin dependent kinase subunit 1 (CKS1) with ubiquitin ligase skp2, preventing ubiquitination and proteasomal degradation of p27. We also detected a p27 upregulation, which is consistent with the hypothesis of Krishnan et al. [33]. In another report using HT29 cells, FLX was shown to inhibit ERK1/2 phosphorylation by hypophosphorylating *c-Myc* and CREB proteins, which resulted in downregulation of cyclin D1 and A, whilst cell-cycle check point genes were upregulated, reducing cell proliferation [20].

Looking at our *in vitro* results and the chemopreventive activity of FLX against MNNG-induced dysplasia through reduced epithelial proliferation, it seems possible that FLX promotes these effects by controlling cell-cycle progression *in vivo*. It is known that cells mutated under MNNG exposure can acquire increased cellular proliferation capacity through changes in oncogene and miRNA expressions [34]. On the other hand, a delay in the G_2_/M phase of the cell-cycle was reported in colon cancer cells exposed to MNNG [35]. Furthermore, the carcinogenic activity of MNNG has been related to high cell proliferation in colonic epithelia [22], [36]–[37], whereas a chemoprevention against the induced dysplastic effects was related to a decrease in proliferation and expression of oncogenes, such as *c-Myc* [36], [38]. Hence, MNNG alters cell-cycle progression through induction of mutations leading to increased proliferative capacity. FLX was found to reduce cryptal proliferation by influencing serotoninergic activity [5] and to induce expression of tumor suppressors, namely p53, p27, and p21 [20], [33].

Since high proliferation plays an important role in angiogenesis [29], we hypothesized that the reduction in stromal proliferation in mice subjected to FLX treatment might played a key role in decreasing the angiogenesis-related dysplasia. Our current findings further support the hypothesis that the anti-angiogenic activity of FLX might be related to the control of angiogenesis-related stem cell markers in colon preneoplastic lesions. Our previous report already showed that FLX reduced colonic microvessel density by decreasing VEGF expression in preneoplastic lesions [5], and that high stromal proliferation increased the number of CD133-positive cells [17]. An intriguing possibility was indicated by the discovery of a small subset of stromal spindle cells expressing CD133 and CD34 in angiofibromas [39]. These authors suggested an acquired potential of stromal cells to transit toward an endothelial phenotype [39]. Further reports reinforced this hypothesis, since endothelial progenitor cells lose the expression of CD133 during their differentiation process into vascular cells, while the expression of CD34 is increased [30], [40]–[41]. This process has also been related to high proliferation [29], which might mean a downregulation in CD34 expression upon further differentiation [42]. Intriguingly, vascular smooth muscle cells increased the expression of CD31 during their differentiation process, whereas a simultaneous decrease of CD133 and CD34 progenitor markers was observed [43]. In addition, CD31-positive cells have been designated as a mature endothelial lineage promoting microvessels [31]. All in all, angiogenesis is one of the most important tissue reactions enhancing preneoplastic lesion formation and tumor growth [14], [29], [44], as well as a suitable target for anti-cancer therapies [45]–[46].

In summary, our findings show that antiproliferative effects of FLX upon colon cells were not dependent on ROS production or DNA damage. Instead, the inhibition of the formation of preneoplastic lesions seems to be achieved by blocking steps of proliferation and angiogenesis-related events in colon tissue. These findings provide new aspects in the understanding of the chemopreventive activity of FLX treatment concerning colon cancer risk.
