# Correction: Antiproliferative Effects of Fluoxetine on Colon Cancer Cells and in a Colonic Carcinogen Mouse Model

**DOI:** 10.1371/annotation/919e5cc0-39bb-4465-babe-d2bdf12e89ff

**Published:** 2013-06-07

**Authors:** Vinicius Kannen, Henning Hintzsche, Dalila L. Zanette, Wilson A. Silva, Sérgio B. Garcia, Ana Maria Waaga-Gasser, Helga Stopper

The following information is missing from the Funding Statement:

"This study was funded by the German Research Foundation (DFG) and by the University of Wuerzburg Open Access Publishing funding program."

